# Authorship Matrix: A Rational Approach to Quantify Individual Contributions and Responsibilities in Multi-Author Scientific Articles

**DOI:** 10.1007/s11948-013-9454-3

**Published:** 2013-06-29

**Authors:** T. Prabhakar Clement

**Affiliations:** Department of Civil Engineering, Auburn University, Auburn, AL USA

**Keywords:** Authorship, Multi-author article, Co-authorship, Author-rank, Authorship-responsibility, Scientific-authoring, Authorship-quantification, Responsible-conduct of research (RCR)

## Abstract

We propose a rational method for addressing an important question—who deserves to be an author of a scientific article? We review various contentious issues associated with this question and recommend that the scientific community should view authorship in terms of contributions and responsibilities, rather than credits. We propose a new paradigm that conceptually divides a scientific article into four basic elements: ideas, work, writing, and stewardship. We employ these four fundamental elements to modify the well-known International Committee of Medical Journal Editors (ICMJE) authorship guidelines. The modified ICMJE guidelines are then used as the basis to develop an approach to quantify individual contributions and responsibilities in multi-author articles. The outcome of the approach is an authorship matrix, which can be used to answer several nagging questions related to authorship.

## Introduction

Who deserves to be an author of a scientific article? Interestingly, scientists and engineers, who have answered many complex questions about nature, have not fully answered this question. This is because the question of authorship involves human elements that cannot be objectively quantified. Therefore, authorship decisions for multi-author publications are usually made using qualitative procedures. This is especially true in applied sciences and engineering fields, where there has been little discussion about authorship policies. Medical and biomedical fields have had multiple debates on this topic that have led to the development of several formal authorship guidelines (ICMJE 2010; Rennie et al. 1997; Savitz 1999). Osborne and Holland (2009) reviewed several of these prominent guidelines and concluded that it is difficult to use a specific guideline to define authorship and author order since each project, team, and discipline would differ in subtle ways.

Cozzarelli, former Editor-in-Chief of PNAS, completed a survey in 2004 and found that the average number of authors on PNAS papers increased from 3 to 7, in about 30 years (Cozzarelli 2004). Such dramatic increases in the number of authors have added more confusion because there are no accepted procedures available for determining author order in multi-author publications; currently, it is decided based on empirical procedures that have little or no basis (Strange, 2008). This practice has several limitations: first, most modern scientific projects require many people to contribute and the magnitude of their contributions could range from trivial to significant. Therefore, determining where and how to draw the line between an author and a non-author is not clear (Savitz 1999). Secondly, based on current practices, it is difficult to interpret the actual meaning intended by author order. Third, there is considerable concern about the practice of “gift authorship,” whereby certain authors are included on the basis of their power and prestige, or based on logistical contributions such as providing funds or granting access to use laboratory facilities, or for providing editorial support. Finally, the current procedures do not emphasize authorship responsibilities, an important issue often ignored by most co-authors (Osborne and Holland 2009; Paneth 1998; Smith and Williams-Jones 2012).

Publishing a paper is an important scientific pursuit; promotion committees and award committees routinely use publications as the ultimate currency to assess the academic value of a researcher. Hilmer and Hilmer (2005) reviewed the annual salaries and publication records of 326 agricultural economics faculty members and concluded that co-authorship and author order affected their salaries. Since authorship could have such high stakes, discussions about author order often lead to serious disputes among scientists. However, there is sufficient evidence in the published literature that the concept of sharing authorship credits based on a specific position on an authorship list is somewhat vague (Strange 2008). It is a common practice to place the name of the person who made the maximum contribution as the first author, and the sequence of co-authors should represent progressively lesser contributions (Huth 1986). However, in several journals (including applied engineering science, chemistry and biology journals) it is customary to place the name of the senior investigator, who might have done considerable work, as last author. Rennie et al. (1997) describes that there is an element of *noblesse oblige* associated with having the stature to be so generous as to place oneself at the end of the list. However, as pointed out by Savitz (1999), rather than downplaying the senior investigator’s role, this practice, at times, is intended to exaggerate one’s own importance. Another authorship practice that is equally confusing is reporting author order in alphabetical order. Disciplines such as mathematics and computer sciences follow this convention (Loui 2006). Since the scientific community does not have a uniform standard to define authorship, the readers currently have no idea about the contributions made by each co-author. Wren et al. (2007) surveyed promotion committee representatives (total of 87 respondents) to assess how contributions of co-authors are perceived by peer groups. Their survey found that the actual contribution of a co-author can differ greatly from the contribution perceived from their byline position. Also, about 40 % of respondents believed that inappropriate granting of authorship is a common practice (Wren et al. 2007).

A possible solution to this problem is to request authors to provide estimates of contribution levels so the readers are fully aware of these details. In the past, several authors have proposed various approaches to quantify contribution levels (Hunt 1991; Tscharntke et al. 2007; Weltzin et al. 2006); however, none of these approaches has gained widespread acceptance. Moreover, the technology required to implement these methods was not developed well in the past. More recently, Frische (2012) identified that online networks can provide the authors of a paper an opportunity to publish detailed accounts of each person’s contribution and argued that it is time to think about full disclosure. The objective of this manuscript is to present a flexible, rational framework which can be used to document the contributions and responsibilities of an author in a multi-author journal article.

## Methods

### What is the Role of an Author?

According to Merriam Webster’s dictionary the word author means: “a person who writes something, or a person who starts or creates something.” Unfortunately, these meanings are of no help when we refer to someone who is listed as an “author” or a “co-author” in a scientific manuscript. The first meaning has no relevance because it is a common practice for non-native English speakers to seek the help of professional editors to edit and write parts of a scientific paper. These professional language experts would never be acknowledged as co-authors.

The second definition: “person who starts or creates something,” also has very little relevance since it is hard to define “something.” Moreover, most research problems are formulated based on past knowledge and it is extremely rare that anyone would “start or create” something totally new in a journal article. Therefore, the dictionary definition of the word “author” provides little or no clarification, and probably adds more confusion.

If we researched the etymology of these words we find that the word author has a Latin root “auctor,” which has a meaning enlarger or literally “one who causes to grow.” The noun form “auctus” is related to “augere” translated into “to increase” and shares the same root as “augment.” These Latin roots imply that one of the major roles of a scientific author is to “contribute” something new to increase our understanding of science. Interestingly, this idea of “contributor” is fully consistent with Rennie et al.’s (1997) bold suggestion that we should abandon the word author in favor of contributor. In this study, we will follow their suggestion and conceptually view authors as contributors of scientific knowledge.

### Responsibility and Authorship

When we use the words “author” or “co-author” to refer to a person associated with a scientific contribution, the words also inevitably imply that this person deserves some sort of credit for the contribution. However, it is important to recognize that the authorship currency has two sides (NRC 2009; Rennie and Flanagin 1994): one is credit, and the other is responsibility. Interestingly, in multi-author papers, the importance of the credit side of the currency is so muddled and overvalued that no one has a clear idea about its real value. Therefore, every author feels free to claim any amount of credit. A multi-author paper may become a bottomless pit full of fortune (i.e., authorship credits) for everyone to enjoy. The responsibility side of the currency is also equally muddled in the other direction. It is so undervalued and diluted that none of the authors feel the need to assume any serious responsibility. Hence, proliferation in the number of authors per article is a win–win solution; no one has to worry about responsibilities and yet everyone can claim any amount of credit. The peer-reviewed literature is full of examples where people who claim credit are quick to abdicate their responsibility when the work was found to be fraudulent or flawed. Strange (2008) reviews a well-known case study, the “Darsee affair,” where Dr. John Darsee who worked at Harvard Medical School and Emory University, published over 18 full-length research papers in the field of cardiology. In May 1981, Darsee admitted to fabricating data in one of the articles and later investigations found several of his other publications also contained fabricated data. Interestingly, several of these publications listed co-authors who have willingly accepted the recognition. When these publications were found to be fraudulent, some of these authors rationalized their role and argued why they are not responsible for the fraud, even though they voluntarily decided to take credit by co-authoring the work. Examples like this show that while many authors are willing to claim credits only a few are willing to share the responsibilities. This case study highlights a fundamental flaw in viewing authorship through the prism of credit.

Therefore, we believe, journal authorship discussions should never involve allocation of credits, a relatively easy part; instead they should focus on allocation of accountability or responsibility, a more difficult part. We will emphasize the later part and define authors as contributors who are willing to take responsibilities for their scientific contributions. However, so far, we have not defined the types of responsibilities associated with scientific authorship. We will define them in the following section using approaches and terminologies used by standard authorship guidelines.

### Review of ICMJE Authorship Guidelines

Currently, there are several policies and procedures available for deciding authorship (ICMJE 2010; Osborne and Holland 2009; PNAS 2013). Among these, the International Committee of Medical Journal Editors’ (ICMJE) uniform requirements for manuscripts submission to biomedical journals is the most accepted guideline, which has been adapted and used by several multi-disciplinary scientific journals (e.g., PLosOne, Science, and Nature). According to these guidelines, authorship credit should be based on: (1) substantial contributions to conception and design, acquisition of data, or analysis and interpretation of data; (2) drafting the article or revising it critically for important intellectual content; and (3) final approval of the version to be published. Authors should meet conditions 1, 2, and 3. These guidelines also state that acquisition of funding, collection of data, or general supervision of the research group alone does not constitute authorship.

These guidelines, however, have several short comings. First, they define authorship in terms of credits. Although the guidelines later state that authors should take public responsibility for appropriate portions of the content, it does not provide a method for quantifying these “appropriate portions.” Secondly, as pointed out by Zbar and Frank (2011), the ICMJE guidelines do not recommend any procedure for determining author order. Finally, these guidelines could potentially disenfranchise senior investigators. Within the ICMJE model, there is no room to give explicit credit to a senior researcher who wrote the proposal that initiated and funded the research effort. Developing research proposals and getting them funded is not a trivial task; especially under current economic conditions where there is an intense competition for dwindling research dollars. Many researchers believe that the ICMJE guidelines are too restrictive and could be out of touch with the challenges of modern science (Bhopal et al. 1997; Smith and Williams-Jones 2012).

### Modified ICMJE Guidelines

We propose minor revisions to ICMJE (2010) guidelines to address some of these limitations. To facilitate these revisions, we prescribe a new conceptual model that views scientific articles in terms of four basic elements for which individual responsibilities can be assigned. Based on this model, a scientific article can be conceptually divided into four basic elements: (1) ideas, (2) work, (3) writing, and (4) stewardship. These basic elements are not new; several authors have made similar suggestions to define various contributions (Osborne and Holland 2009; Paneth 1998). In this study, the element “ideas” will be used to signify all intellectual contributions. The element “work” will be used to signify the efforts invested by the team for collecting, analyzing, and interpreting data. The element “writing” will be used to signify the efforts invested for writing. And, finally, the element “stewardship” will be used to signify the efforts invested to develop and direct the work. Stewardship is about reflecting on the research problem well before the work was initiated and continuing to own the problem well after the work was published. It is about someone willing to invest the time to develop resources, provide long-term leadership, and be the guarantor of the entire work.

We will employ these four basic elements to revise the ICMJE guidelines, and emphasize the responsibility side of the authorship equation to define the role of an author. An “author” or a “co-author” of a journal article is:

1. responsible for conception of the problem, theorizing and designing experiments, and/or interpreting data (contributions to “ideas”);
2. responsible for data acquisition, data analysis, coding, and/or using the instruments/models (contributions to “work”);
3. responsible for drafting the article or revising it critically for important intellectual content and approving the final version (contributions to “writing”); and
4. responsible for developing resources, and guaranteeing the overall integrity of the work as a whole before and after publication (contributions to “stewardship”).

Following ICMJE’s approach, we expect every author to contribute sufficiently to the entire research, and take a reasonable level of responsibility for three of these four basic elements. Every co-author must contribute to ideas and writing, the two key elements. In addition, every co-author should also contribute either to work or to stewardship. We recommend contributing to a total of three, instead of all four, since junior authors, such as graduate students and post-docs, might not have an opportunity to contribute to the fourth element. Also, some senior authors might not be involved in daily data collection efforts, but still could be fully engaged in the project on a continuous basis. It is important to recognize that continuous engagement (not just providing some brief technical suggestions) is one of the key hallmarks of authorship.

Based on these revised guidelines, an author, by definition, is a person who is fully engaged in the project on a continuous basis and is willing to take responsibilities for three of the four basic elements. While these revised guidelines provide a qualitative framework to define an author, they do not provide answers to several specific questions such as: who is an author and who is not? What is the meaning of author rank? What is the level of responsibility owned by each co-author? To answer these questions we need a rational quantitative framework that can be used to assess the contributions made by each author.

### Matrix Method for Quantifying Authorship Contributions and Responsibilities

In this section, we will adapt the revised ICMJE guidelines to develop a method to quantify authorship contributions. The rationale for developing this methodology stems from the need for a method for estimating individual contribution levels (and the associated responsibilities), which are needed to objectively rank authors in a multi-author paper. In the published literature, researchers have attempted to develop several empirical and semi-quantitative approaches for quantifying authorship contributions (Hunt 1991; Oberlander and Spencer 2006; Resnik 1997; Sheskin 2006; Tscharntke et al. 2007; Weltzin et al. 2006). However, none of these methods has gained widespread acceptance. Also, none of these approaches has been integrated with a standard authorship guideline.

The quantitative methodology proposed here, named as the authorship matrix method, is fully integrated with the ICMJE guidelines. At the core, the method employs two fundamental matrices to quantify individual responsibilities. It is a cascading scheme; at a minimum, the method requires an element-allocation matrix and an authorship contribution/responsibility matrix. If needed, each element can be further divided into sub-elements to build element-specific contribution matrices. Note that the term “matrix” is used to keep the definitions succinct and there is no need for any matrix mathematics to understand or use this methodology. This quantitative method requires three sequential steps that can be easily implemented within any standard spreadsheet tool such as EXCEL. In sections below we will use a case study to demonstrate these three steps and illustrate their use.

## Case Study

### Step 1: Development of Basic Manuscript Element Allocation Matrix

The first step in using the proposed method is to divide the efforts invested for developing a scientific manuscript into the four basic elements: ideas, work, writing and stewardship. We will use a hypothetical example to illustrate this step. The example considered is roughly based on a journal article that our group recently developed (a few details are modified and fictitious names are used). The objective of the project was to characterize Deepwater Horizon oil spill (BP spill) related wastes collected from several beaches in Alabama. The team consisted of five members: a second year graduate student (Jim Johnson), a lab technician (Jack Norman), a post-doctoral fellow (Jill Jones), an associate professor (Janet Fonda), and a full professor (Clem Peter). Professors Peter and Fonda jointly developed the grant proposal that funded the effort. The project involved collection of field samples, analysis of the samples to quantify polycyclic aromatic hydrocarbons, analysis of the data, preparation of figures, and writing the manuscript. The lab work primarily employed standard techniques, but a new instrument, which required considerable tuning and calibration, was used to characterize several unique samples collected by the field team. After completing the first draft of the manuscript, the group discussed and divided the overall effort into: 0.2, 0.3, 0.35, 0.15 (or 20, 30, 35, 15 %) for the four basic elements ideas, work, writing and stewardship, respectively. These values were recorded in a spreadsheet tool (EXCEL) as weight fractions, instead of percentages, purely for computational convenience (see Table 1).

**Table 1:** Basic manuscript element matrix

Ideas	Work	Writing	Stewardship	Total
0.2	0.3	0.35	0.15	1

It is important to note that these weight fractions are not fixed estimates; they can be dynamic and can vary for different projects. As the manuscript evolves, one could potentially revisit these numbers and adjust them, if the group felt it is needed. However, in practice, one would quickly realize that there is not much room for adjustments since the four element fractions should add to unity, an extremely useful mathematical constraint. Keeping track of such simple mathematical constraints is an incredibly powerful way to manage this problem and we will illustrate its unique power in the next section. Also, one could impose several other meaningful constraints (discussed below) on the relative weights of various elements that would thwart any serious revisions.

First, it is important to carefully assess the weight assigned to the first element “ideas.” Several ideas, especially the ones suggested in open forums such as group meetings or seminars, are primarily shared as technical suggestions; and no one really knows whether these suggestions are feasible ahead of time. Even great ideas require considerable amount of work and writing to bring to fruition. Therefore, in most cases, the weight assigned to ideas should be less than the weight assigned to work and writing which are the two most important elements. However, this is not a rule; it is just an empirical suggestion.

Secondly, the fourth element “stewardship” should always get the lowest weight since it only makes indirect contributions. This, perhaps, should be considered as a rule. It is important to note that most guidelines, including the original ICMJE (2010) method, do not give any credit for resources, funding, and research management. The proposed approach is flexible to accommodate these conventional guidelines; if one chooses to follow them, they should simply assign zero to the stewardship element.

With some of these constraints in mind, good initial estimates for the four basic elements are: 0.25, 0.30, 0.30, and 0.15. These are fairly realistic estimates for any standard article where data and writing will be the two most important elements. With these initial estimates, any team can discuss and calibrate the values to fit the needs of their own manuscript. It is important to include all potential authors in these discussions. Also, it is important to communicate with others in the team why they will not be recognized as co-authors. When we exclude potential co-authors we have an obligation to let them know about our decision well ahead of time. This is an excellent approach to avoid authorship conflicts.

### Step 2: Development of a Draft Authorship Matrix

The second step is to develop a draft version of the authorship matrix. This step is an iterative process where the goal of the first iteration is to tease out all underlying problems related to authorship sharing. Step-2 should be started as an individual assessment process. The senior author (person who is willing to be the steward or guarantor of the work, it was Peter in this case study) should communicate with all potential authors and ask them to estimate their contribution levels. Jim Johnson (graduate student) is the youngest author in our group who did most of the field work, analyzed various samples, and also contributed to writing. When Peter informally asked him to estimate his contributions, Jim was quick to say that he appreciated others help, but he did most of the work and hence should get at least 50 % of credits for this paper. Note he automatically focused on credits, a natural instinct. As a second step, Peter sent a follow-up email reminding him about his estimate of 50 % and asked him whether he could itemize his contributions to each of the four basic elements. Jim replied quickly and his estimates were 30, 80, 60, and 10 % for ideas, work, writing and stewardship, respectively. These numbers were entered into the draft matrix and was weighted based on respective element fractions and the net contribution made by Johnson was estimated to be 52.5 % (the actual computation is: 30 %*0.2 + 80 %*0.3 + 60 %*0.35 + 10 %*0.15 = 52.5 %). Interestingly, the itemized estimates yielded a value fairly close to his rough estimate of 50 %. The above process was repeated for all five researchers and the estimates are summarized in Table 2.

**Table 2:** Draft authorship matrix

	Ideas	Work	Writing	Stewardship	Net contribution/responsibility (%)
	0.2 (%)	0.3 (%)	0.35 (%)	0.15 (%)	
Johnson (graduate student)	30	80	60	10	53
Norman (technician)	20	50	10	15	25
Jones (post-doc)	40	60	70	20	54
Fonda (associate professor)	40	0	25	40	23
Peter (professor)	60	30	60	70	53
Balance check	190	220	225	155	206

The first observation to be made from these data is that the three active contributors (Johnson, Jones and Peter), who were fully engaged in the project, felt that they contributed to about 50 % of the project. The other two passive contributors (Norman and Fonda) felt that they contributed about 25 %. Based on several such surveys we have found that these are typical estimates most researchers (both young and experienced) would assign when their names appear on a multi-author article with about five to six co-authors. The problem is that these estimates violate “mathematical balance” constraints! Note, in Table 2, the sum for each column is well over 100 %, with a net total of 206 %; this implies that the group has contributed to two articles! This sort of overestimation routinely occurs in the current model when each author claims his/her own share of credit on their CV, annual review, or promotion application. We just do not have an unbiased approach to document this overestimation problem. This problem occurs because we currently do not have a process to evaluate the relative worthiness of our contribution in light of other’s contributions. For example, after reviewing Table 2, graduate student Johnson quickly realized that when he claimed that he did 80 % of work he also implicitly judged that his four colleagues, who were also equally engaged in the project, did only 20 % of the work, which was not true in this project effort. One of the useful outcomes of the proposed method is that it will help demonstrate such inconsistencies, which can be used to discuss the importance of team work and compromise. Having this information on paper will help educate the team to develop a sense of mutual respect and camaraderie.

### Step-3: Finalizing the Authorship Matrix

The next step is to discuss these draft estimates and negotiate as a group to balance all four elements. The best approach is to adjust each element (or column) to satisfy the associated mathematical balance constraint. If required, one could complete a more detailed assessment by further dividing the element of interest into sub elements. Such divisions could be particularly useful for complex elements such as “work,” which might involve multiple tasks. The power of the proposed framework is that it can be refined into a cascading set of sub-matrices to analyze such complex elements. To demonstrate this process, first we will divide “work” into three sub-elements. Note one could use any number of sub-elements; here we divided work into the following three sub-elements: field work, lab work, and data-analysis work. We then followed a step similar to Step-1 to estimate the fractional weight for each of these work elements and these estimates are summarized in Table 3. The next step is to create a work-load allocation matrix (which is similar to Step 2) where each individual researcher was asked to estimate his/her own contribution to all three work elements. These estimates were agreed upon as a group and weighted with appropriate fractions to develop a balanced work allocation matrix shown in Table 4. We can now use these balanced estimates to revise the work element in the authorship matrix.

**Table 3:** Basic work element matrix

Field work	Lab work	Data analysis	Total
0.1	0.6	0.3	1

**Table 4:** Work load allocation matrix

	Field work	Lab work	Data analysis	Work (%)
	0.1 (%)	0.6 (%)	0.3 (%)	
Johnson (graduate student)	70	55	50	55
Norman (technician)	10	15	0	10
Jones (post-doc)	0	30	30	27
Fonda (associate professor)	0	0	0	0
Peter (professor)	20	0	20	8
Balance check	100	100	100	100

Note that the development of sub-matrices (shown in Tables 3, 4) is an optional step, which can be skipped if one can directly formulate the final version of the responsibility matrix by negotiating the numbers as a team to satisfy all the balance constraints. In this case study, we adjusted the “work” element using the cascading sub-matrix method, whereas the other three elements (ideas, writing and stewardship) were adjusted by direct negotiations. The final version of the full authorship matrix is shown in Table 5.

**Table 5:** Final authorship matrix

	Ideas	Work	Writing	Stewardship	Net contribution/responsibility (%)
	0.2 (%)	0.3 (%)	0.35 (%)	0.15 (%)	
Johnson (graduate student)	5	55	35	0	29.8
Norman (technician)	15	10	5	10	9.3
Jones (post-doc)	25	27	35	10	26.9
Fonda (associate professor)	15	0	5	30	9.3
Peter (professor/guarantor)	40	8	20	50	24.9
Balance check	100	100	100	100	100

The authorship matrix provides all necessary information for deciding the rank of an author. Based on these data, Johnson will be the first author, Jones the second author, and the senior author Peter will be the third author or he could invoke the practice of *noblesse oblige* and serve as last author, depending on his personal preference. If there is a tie, we believe senior personnel (in this case Fonda) should be placed behind the junior author. This matrix methodology solves the author order problem without any ambiguity. The person who has contributed to all four elements and has considerable responsibility for the element “stewardship” (Peter, in this case) should serve as the guarantor for the article. He/she should also oversee the construction of authorship matrix and communicate with all potential authors about their respective responsibility levels.

The final issue to be addressed is what should be the minimum total contribution (MTC) below which one would not qualify for recognition as a co-author. A good rule of thumb to guide this process is every co-author should contribute a minimum of 50 % of the average contribution. This rule can be defined using the mathematical formula:

$$ {\text{Minimum}}\,{\text{Total}}\,{\text{Contribution}}\,\left( {\text{MTC}} \right) = \left( {0.5*100\% } \right)/{\text{n}} $$

where n is the total number of qualified co-authors who have contributed for three or more basic elements. The important point here is that the cut-off level should depend on the total number of authors; for example, while it is reasonable to contribute 5 % on a large collaborative ten author article, the same 5 % might not be adequate if it is a focused article with just two authors. The proposed rule of thumb helps capture this relationship. Using the above formula, MTC cut-off level will be 16.7 % for three authors (n = 3); 10 % for five authors (n = 5), and 5 % for 10 authors (n = 10). Since these are rules of thumb estimates, some level of personal judgment should be exercised when using these estimates. The cut-off MTC level for the current case study is 10 %; both Norman and Fonda have contributed 9.3 %, which is quite close to the cut-off level and hence should qualify for co-authorship (note, one of the senior author’s numbers can be adjusted to maintain the overall balance). The final authorship data can be presented in journals either in the detailed matrix format shown in Table 5, or can be rounded off and condensed into a concise summary format shown in Table 6.

**Table 6:** Authorship contributions

Johnson	Norman	Jones	Fonda	Peter
30 %	10 %	27 %	10 %	23 %

## Discussion

In this work, we propose a rational framework for solving the authorship dilemma. The proposed solution is by no means a panacea since the scope of this effort is limited to standard, full length research articles that require years of planning and work, and multiple contributors. This effort is only a small step towards solving a difficult problem that is intricately integrated with human emotions. Rennie et al. (1997) warned that any attempt to quantify contribution levels could lead to hair-splitting negotiations about the origin of ideas and the amount of work done. We fully agree and acknowledge these risks; but we also believe that potential benefits will far outweigh these risks. Moreover, it is a timely effort since several databases have already started to introduce new metrics based on a scientist’s contributions and activity data available in the network (Frische 2012). Therefore, journals should at least provide authors an opportunity to publish detailed data of each person’s contribution so the readers know what each one has contributed. This information can be used to develop transparent merit lists that can be openly validated by all participants (Frische 2012).

The data presented in the authorship matrix, for example, can be used to develop other metrics such as contribution-adjusted productivity (CAP) index. If a researcher primarily worked in large groups and published five multi-author papers with an average contribution level of 10 % in each article, then his CAP will be 0.5 (=5*0.1). Note the CAP of this researcher will be equivalent to a researcher who worked with his colleague (or student) and published just one 2-author article, for which he has contributed 50 % (CAP = 1*0.5 = 0.5).

These contribution-adjusted credits can be further weighted by other parameters such as journal impact factors to derive more effective parameters that can serve as better metrics for assessing research productivity. For example, one can compute an impact-factor weighted CAP (ICAP) for a person who worked on a large project and contributed about 5 % to a Nature article (impact factor of 36) with ten co-authors as 1.8 (=0.05*36). On the other hand, the ICAP for a person who worked closely with a student and made 45 % contribution to a two-author PlosOne (impact factor of 4) article will also be 1.8 (=0.45*4). Such scaled estimates, which can be derived from the data provided in an authorship matrix, will be extremely useful for comparing the relative worthiness of research outputs.

The authorship matrix data can also be used by citation databases to distribute citation credits. Currently, every co-author gets the exact amount of citation credit regardless of their author order (or contribution). This practice violates mathematical balance (since multi-author papers award full citation credit for multiple researchers for publishing a single article); and it inflates the citation record of researchers who work on multi-author articles. This also diminishes the true value single-investigator or student-supervisor contributions. If an authorship matrix is made available to citation databases, one can compute a contribution-adjusted citation (CAC) index that can more effectively normalize the citation record. For our example problem, if the Nature article received 100 citations, CAC assigned for the Nature author will be 5 (=100*0.05); interestingly, the PlosOne author has to receive only about 11 citations to get a similar level of CAC (11*0.45 = 4.95).

These are just a few example metrics that can be easily derived from authorship matrix data. We acknowledge that deriving such metrics could play into the mindset of trying to quantify scholarship in terms of highly simplistic numbers. But we must be wary that such arguments have been made in the past (and continue to be made in some traditional disciplines) to argue against the use of standard metrics such as citations and h-index. Similar to h-index, the indices proposed here are just simple surrogates for quantifying scholarship, and they are no means substitutes for contextual sensitivity and personal judgment.

We propose that journals should at least encourage authors to develop and publish a summary matrix (shown in Table 6), along with some qualitative descriptions explaining who contributed what. On the other hand, if the full matrix was published (Table 5), individual contributions become self-explanatory and hence there is no need for any qualitative statements. When the final revised version of the manuscript is accepted, publishers could request the guarantor to upload a signed copy of an authorship matrix with an agreement that they have been reviewed and approved by all co-authors. The guarantor should handle all these communications and he/she will have both legal and moral responsibility for resolving any future conflicts related to responsibilities. Journal editors should simply redirect any future queries to the guarantors and let them work with their parent institutions and funding agencies to deal with any post-publication issues and send them a final report. Universities, research organizations, and funding agencies should develop their own policy procedures to deal with such post-publication conflicts. For example, there could be a post-publication grievance committee, operated by the University Senate or other such internal bodies, which can develop ethical policies for handling all post-publication conflicts related to authorship responsibilities and credits.

We propose that every multi-author article should clearly identify a guarantor. The guarantor should be recognized as a significant contributor and he/she must be fully prepared to defend all parts of the manuscript before and after publication. According to Rennie et al. (1997), guarantors should have not only contributed substantially to the article, but they also should have made added efforts to organize, oversee, and double-check to ensure the integrity of the entire project. While some journals such PNAS allow authors to identify multiple guarantors (Cozzarelli 2004; PNAS 2013), we propose that standard research articles (e.g., articles with about five authors or below) should identify a single guarantor to avoid any further dilution of this important responsibility. However, there could be some exceptions for multi-disciplinary projects or reviews that require large groups of authors with diverse expertise. It is highly likely that one of the senior personnel, who initiated the work, secured funding, and led the research team would naturally fit the guarantor role. In rare cases, the lead author can serve as guarantor if he/she was part of the efforts that initiated the project, and has the desire to serve as the long-term steward of the work.

The current practice of “corresponding author,” used in many journals, is yet another confusing terminology (Cozzarelli 2004). Journal policies should clarify the role of corresponding authors and clearly indicate whether they would shoulder the responsibilities of guarantor or not. By identifying a single point of contact (designated either as guarantor or corresponding author) the readers would know the author who is taking moral responsibility for the work as a whole.

It is important to note that the development of an authorship matrix is a dynamic process. Preparation of a journal manuscript is an unfinished work of art that will be constantly worked on until the manuscript gets published. Therefore, the numbers agreed upon in the beginning should be reconsidered periodically as the roles of authors change. Baughman (1999) pointed out that in any collaborative work, some roles may be diminished while others may be enhanced over time. He provides an excellent example where a manuscript rejected by a journal might be left unrevised for years until a coauthor (or even a non-author colleague) might take the initiative to finish the manuscript. Therefore, it is important to revisit the authorship matrix periodically to revise these numbers, especially when there are changes in personnel and/or scope of work. These numbers cannot be set in stone; they should be viewed as dynamic estimates that will be updated periodically by the team until the manuscript is published.

One of the most important benefits of the proposed approach is that it helps develop an open forum to discuss various contentious authorship issues on a regular basis. Such a forum can help promote an environment where team members can be educated to self-evaluate the relative merits of their contribution in light of others’ contributions over time. The mathematical balance constraints are particularly useful and can help avoid questionable practices such as guest authorship. For example, in our case study, if we attempted to include a guest author Robert Paul (department chair who provided funds for equipment), we have to assume some numbers to quantify Paul’s contribution; but these contributions have to come from somewhere and we will be forced to rob Peter (and his team) to acknowledge Paul. The numbers would clearly show that we are not only giving undeserved credits to our guest author, but also taking away well-deserved credits from legitimate authors who did years of work.

We fully acknowledge the fact that conflicts regarding ownership and credits can never be fully avoided (Resnik 1997); however, we believe they could be either minimized or resolved in an amicable manner if the proposed framework is used within an environment of mutual respect. Besides all these pedantic assessments, an important empirical test for deciding authorship is the following: regardless of any quantitative metric, every co-author should be able to give a short presentation describing their contributions within the intellectual context of the manuscript at any time before and after publication (Strange 2008). This is the ultimate test for authorship.

## Concluding Remarks

In this paper we address a fundamental question relevant to every researcher who will ever consider publishing an article: what is the meaning of the term author in a scientific publication? We suggest that the scientific community should conceptually view journal authors as contributors who assume certain responsibilities. We propose a rational approach to quantify the responsibilities of an author of a scientific manuscript and use the data to objectively answer the authorship question. It is important to note that our goal here is not to solve the complex authorship problem entirely; rather the goal is to advance the debate in a small way and let journals, professional organizations, research institutions and individual researchers discuss and develop their own set of policies.

The proposed solution, however, has many limitations. One of the major concerns is that any attempt to quantify contributions using detailed numbers might lead to contentious arguments (Loui 2006; Rennie et al. 1997). This is a legitimate concern; however, at a minimum, the proposed framework could be used to facilitate open discussions to directly develop the summary matrix (Table 6) and some qualitative statements explaining individual contributions. Several medical journals and other well-known journals such as PNAS, PLOS One, Nature and Science are already requiring authors to include qualitative statements. Drummond Rennie, who has served as the deputy editor of New England Journal of Medicine and Journal of the American Medical Association, reviewed this practice and concluded that the simple act of explicitly stating what individual authors claim to be their contributions would considerably increase author accountability. He went on to recommend that all journals should require such statements (Rennie 2010). We couldn’t agree with him more; at a minimum, journals should publish statements of responsibility. He also pointed out that the scientific community should adhere to a minimum set of universally accepted criteria for authorship (Rennie and Flanagin 1994), and we believe the revised ICMJE guidelines proposed in this study is a good starting point for all journals.

Finally, whether we use the term author or contributor might not be all that important; the key is to change the paradigm and view authorship in terms of accountability rather than credits. We have attempted to introduce this change in the current effort. The philosophical underpinnings of the proposed approach are elegantly summarized by the following quote attributed to Richard Hewitt, the former head of Publication Section at Mayo Clinic (Huth 1982): “*authorship cannot be conferred: it may be undertaken by one who will shoulder the responsibility that goes with it. To a responsible writer, an article, with his name on it, is the highest product of his mind and art, his property, as nearly flawless as he can make it, founded in his character and evidence of it. If that describes the acceptable standard, (medical) writers, a responsible group, are in present need of reconsidering the implication of joint authorship. The reader of a report issued by two or more authors has a right to assume that each author has some authoritative knowledge of that subject, that each contributed to the investigation, and that each labored on the report to the extent of weighting every word and quantity in it.*” These profound statements succinctly summarize the true meaning of authorship.
